# Design of Multifunctional Titania-Based Photocatalysts by Controlled Redox Reactions

**DOI:** 10.3390/ma13030758

**Published:** 2020-02-07

**Authors:** Diogo Lopes, Ana Luísa Daniel-da-Silva, Artur R. Sarabando, Blanca I. Arias-Serrano, Elena Rodríguez-Aguado, Enrique Rodríguez-Castellón, Tito Trindade, Jorge R. Frade, Andrei V. Kovalevsky

**Affiliations:** 1 CICECO–Aveiro Institute of Materials, Department of Materials and Ceramic Engineering, University of Aveiro, 3810-193 Aveiro, Portugal; djlopes@ua.pt (D.L.); blanca@ua.pt (B.I.A.-S.); jfrade@ua.pt (J.R.F.); 2 CICECO–Aveiro Institute of Materials, Department of Chemistry, University of Aveiro, 3810-193 Aveiro, Portugal; ana.luisa@ua.pt (A.L.D.-d.-S.); tito@ua.pt (T.T.); 3 Departamento de Química Inorgánica, Cristalografía y Mineralogía (Unidad Asociada al ICP-CSIC), Facultad de Ciencias, Universidad de Málaga, Campus de Teatinos, 29071 Málaga, Spain; aguadoelena5@gmail.com (E.R.-A.); castellon@uma.es (E.R.-C.)

**Keywords:** photocatalysis, TiO_2_, anatase, silicon carbide, Taguchi plan, redox reaction

## Abstract

This work aims at the preparation of multifunctional titania-based photocatalysts with inherent capabilities for thermal co-activation and stabilisation of anatase polymorph, by designing the phase composition and microstructure of rutile-silicon carbide mixture. The processing involved a conventional solid state route, including partial pre-reduction of rutile by SiC in inert Ar atmosphere, followed by post-oxidation in air. The impacts of processing conditions on the phase composition and photocatalytic activity were evaluated using Taguchi planning. The XRD studies confirmed the presence of rutile/anatase mixtures in the post-oxidised samples. The results emphasise that pre-reduction and post-oxidation temperatures are critical in defining the phase composition, while post-oxidation time is relevant for the photocatalytic performance. Microstructural studies revealed the formation of core-shell particles, which can suppress the photocatalytic activity. The highest apparent reaction rate of the photodegradation of methylene blue was observed for the sample pre-reduced in Ar at 1300 °C for 5 h and then calcined in air at 400 °C for 25 h. Though its performance was ~1.6-times lower than that for the same amount of nanostructured industrial P25 photocatalyst, it was achieved in the material possessing 2–3 times lower surface area and containing ~50 mol% of SiO_2_ and SiC, thus demonstrating excellent prospects for further improvements.

## 1. Introduction

Vast research interest in the development of titanium-oxide-based photocatalysts is encouraged by the great capacity of these materials for boosting various important reactions and combination of chemical stability, abundance, biocompatibility and nontoxicity [1]. Prospective applications include water and air purification, photocatalytic water splitting for hydrogen production, self-cleaning surfaces, disinfection, photo-promoted bone-implant fixation, etc., with enormous potential for various hybrid concepts. Since one of the first work dated back to 1964 [2], demonstrating the oxidation of tetralin in the liquid phase promoted by TiO_2_ under UV light, many research efforts were focused on effective material design approaches to increase the photocatalytic activity and to shift the bandgap to visible light absorption by doping, surface modifications, tuning anatase-rutile phase transitions, and creation of various multicomponent heterojunctions [3].

Progress in the development of efficient photocatalytic materials is largely correlated with the advances in nanotechnology [1,4]. A majority of the reports on advanced TiO_2_-based photocatalysts deals with the nanosized materials. Still, the health aspects of the entire class of nanomaterials are debatable, especially in terms of long-time-exposure effects [5]. Microscale platforms may also provide significant advantages in terms of photocatalyst recovery. The fact that the nanosized TiO_2_ usually offers an improved performance over microscale particles [6], however, does not exclude advantages of microstructured TiO_2_-based photocatalyst in specific scenarios, namely where the larger size may facilitate the photocatalyst separation, increase a dispersibility for potential long-range transport [7], and avoid erosion by a high medium flux. One should also account for the costs and environmental impacts associated with the production of nanomaterials, as compared to micropowders. Several research works are showing that microsized TiO_2_ can successfully replace the nanometric powders in photocatalytic applications [7,8,9], also within the concepts involving further decoration with other nanostructures [10]. In the above works, the studies were performed using a commercial TiO_2_ powder. However, along with seeking potential applications where the advantages of microsized photocatalysts outweigh their disadvantages such as an inherently low surface area [7,8,9,10], one should take into account that the microscale platforms also offer additional possibilities for materials design. In particular, the morphology and phase composition of the microparticles can be tuned by the treatments under elevated temperatures without undue risk of their coarsening. Secondly, the diffusion length in such materials is not constrained by nanodimensions; this opens new pathways for their tailoring by controlled interactions in composite formulations and by the processing involving interaction with the atmosphere. Thirdly, the micropowders can be further modified to bear surface nanostructuring, with great potential to impart various functionalities. To our best knowledge, most of these possibilities and their potentially synergistic combinations were not yet demonstrated even for TiO_2_, a widely used photocatalyst material.

In addition to size-scale-related aspects, the photocatalytic performance of titania is largely determined by the phase composition, with emphasis on the anatase and rutile polymorphs [11]. Various additives can be also used to tune the band-gap, improve the charge carrier separation, and affect the adsorption of various species at the surface [12,13,14,15,16,17]. In general, the anatase is considered as the most active phase towards photocatalysis [11,18]. Although the reasons for that are still debatable, this fact can be likely attributed to a longer lifetime of photoexcited electrons and holes in anatase and differences in bulk charge carrier mobility. Some works point out that the anatase/rutile mixture may even outperform the single anatase phase. As an example, widely used commercial P25 photocatalysts are composed of both phases. A partial reduction of Ti^4+^ cations was found beneficial to boost absorption in the visible light spectrum [19,20]; while the presence of Magnéli phases in anatase significantly promotes the photocatalytic activity [21]. Thus, a promising research direction may rely on the possible synergies provided by the simultaneous tuning of the phase composition and nanostructuring combined with a suitable redox design, aiming to produce efficient TiO_2_-based photocatalysts. 

This work demonstrates a new microscale material design approach to process redox-transformed TiO_2_ + SiC mixtures as suitable photocatalysts. The choice of silica as a secondary phase is determined by its inhibitory effect on the anatase transformation [22,23] and also because it is one of the main components of conventional ceramics. The presence of silicon carbide (SiC) may allow the self-heating of the catalyst by microwave radiation, combining the photocatalytic activity with a thermal co-activation functionality [24]. One takes advantage of the Taguchi experimental planning for the rational design of produced redox-tuned composites towards optimised phase composition and enhanced photocatalytic performance.

## 2. Materials and Methods

### 2.1. Processing of the Photocatalysts

**Figure 1:**
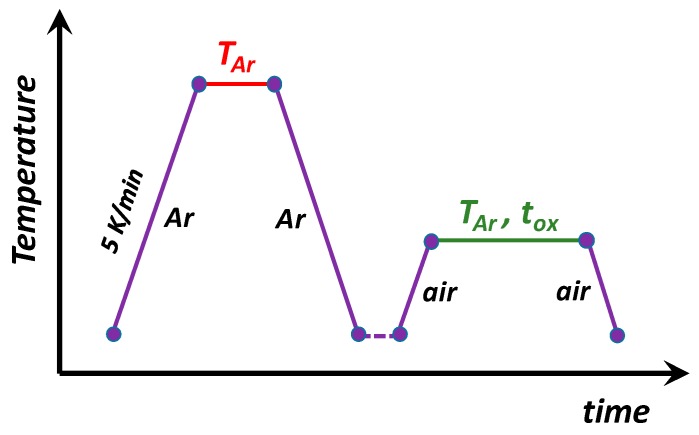
Qualitative diagram of the thermal treatment procedure used for the preparation of the redox-tuned samples.

The precursor powders for the preparation of the target composite materials included titanium (IV) dioxide, rutile (TiO_2_, Alfa Aesar, Kandel, Germany) and silicon carbide, β-phase (SiC, Alfa Aesar, Kandel, Germany). These powders were mixed in a molar proportion of 1:1, and ball-milled with ethanol, using a nylon container with Tosoh tetragonal zirconia milling media, for 4 h at 50 rpm. After drying, the mixed powders were used for the preparation of ~10 mm-thick pellets by uniaxial pressing at ≈ 40 MPa during ~30 s. After compacting, the pellets were pre-reduced in Ar atmosphere and further post-oxidised in air to assess the possibility for the formation and maintaining various titania-based phases for improved photocatalytic activity. A Taguchi experimental planning was then implemented in order to understand the effect of the processing conditions on the phase composition and photocatalytic activity. The plan involved three variable parameters, namely, the pre-reduction temperature in Ar (T_Ar_), and the post-oxidation temperature (T_ox_) and time (t_ox_). This route is shown in Figure 1.

The time scale for post-oxidation was selected assuming typical logarithmic dependence of the oxidation rate on time. Table 1 and Table 2 show the levels and orthogonal array for the corresponding processing conditions.

Table 3 lists the notations and preparation conditions for other samples, used for comparative studies.

### 2.2. Materials Characterisation

After the post-oxidation step, all samples were converted to the powders (except for the microstructural characterisation of the ceramic samples). The XRD phase analysis was performed using a high-resolution diffractometer (Malvern PANalytical X’Pert, Worcestershire, United Kingdom) using Cu K_α_ (I = 1.540598 Å) radiation. The data acquisition was performed for 2Θ from 5 to 80^°^, with ½ and ¼ slits and a 200 s step time. The quantitative content of the crystalline phases was obtained using the Rietveld refinement method (TOPAS Version 4.2, Bruker AXS, Karlsruhe, Germany).

The combined SEM/EDS studies were performed using both fractured ceramic samples and powders on a Hitachi SU-70 instrument equipped with a Bruker Quantax 400 detector. X-ray photoelectronic spectroscopy (XPS) measurements were carried out on a Physical Electronics spectrometer (PHI Versa Probe II) with monochromatic X-ray Al Kα radiation (100 µm, 100 W, 20 kV, 1486.6 eV) and a dual-beam charge neutralizer. The energy scale of the spectrometer was calibrated using Cu 2*p*_3/2_, Ag 3*d*_5/2_, and Au 4*f*_7/2_ photoelectron lines at 932.7, 368.2, and 84.0 eV, respectively. Under a constant pass energy mode at 23.5 eV condition, the Au 4*f*_7/2_ line was recorded with 0.73 eV FWHM at a binding energy (BE) of 84.0 eV. The collected XPS spectra were analysed using PHI SmartSoft software and processed using MultiPak. The binding energy values were referenced to adventitious carbon C 1*s* signal (284.8 eV). Recorded spectra were always fitted using Gauss–Lorentz curves. 

Nitrogen physisorption experiments were performed to investigate the specific surface area and porosity of the materials with a Gemini V2.0 Micromeritics Instrument (Micromeritics, Norcross, GA, USA). The specific surface area was calculated from the N_2_ sorption data using the BET (Brunauer–Emmett–Teller) equation [25]. The total pore volume (V_T_) was defined as the volume of liquid nitrogen corresponding to the amount adsorbed at a relative pressure P/P_0_ = 0.99 (Gurvitch rule) [26].

### 2.3. Photocatalytic Experiments

The photocatalytic tests were conducted using a borosilicate glass jacketed reactor (capacity 250 mL) with a photochemical quartz immersion well (Ace Glass Incorporated). The quartz immersion well, was a double-walled, with inlet and outlet tubes for cooling. A bottom flat reactor was used in order to allow magnetic stirring for keeping relatively large powder particles in the suspension during the test. Methylene blue (MB) (Riedel-de Haen, methylenblau B extra for microscopy) was used as a model organic dye to study the photodegradation. The reference photocatalytic powder, 80% anatase and 20% rutile (Aeroxide, TiO_2_ P25, Evonik Industries, Hanau, Germany) was also tested to compare the performance with the studied materials. The tests were performed with the initial MB solution concentration of 10 mg/L. The volume of solution for the reaction used was 200 mL and the amount of photocatalytic powder used was 0.1 g in all cases, including P25. All tests included the first hour in the dark in order to establish the adsorption/desorption equilibrium of MB molecules onto the material’s surface. After that, the suspension was irradiated by a Hg lamp (SUPRATEC HTC 150-211 UV lamp, supplied by Osram) with a power of 22 W for UVA wavelength (315–400 nm) and 6 W for UVB wavelength (280–315 nm). The reaction mixture was kept at 25 °C. The aliquots (4 mL each) were taken every 10 min and centrifuged for 5 min (14000 rpm) to remove the powder before further analysis. The absorbance of the supernatant was analysed with an ultraviolet-visible spectrophotometer (GBC, Cintra 303). The acquisition of the data was done using the software Cintral, and the conditions used included a scan interval of 800 to 300 nm, with a rate of 200 nm/min, a step size of 0.480 nm and a slit width of 2.0 nm. The reference used was deionized water. The concentration of methylene blue in aliquots taken was calculated based on the Beer–Lambert law by measuring the absorbance at 665 nm.

## 3. Results

### 3.1. Processing Effects on the Phase Composition

The selected processing route for the preparation of titania-based composites included the powder pressing as one of the steps, and further sintering of the ceramic samples. From the first look, this approach does not appear to be optimal for processing materials suitable as photocatalysts, where nanostructuring and a large available surface area are usually required [27]. However, the selected method allowed to provide better contact between the rutile and SiC particles during the pre-reduction reaction and to minimize surface oxidation of silicon carbide without reaction with TiO_2_. To avoid ambiguities arising from intermediate ceramics grinding, both steps of processing in quasi-inert Ar atmosphere and post-oxidation were done for ceramic samples. Preliminary tests showed that the selected range of processing conditions does not result in excessive densification (the ceramic samples were porous enough to soak water). Thus, sufficient open porosity of the pre-reduced samples was provided.

**Figure 2:**
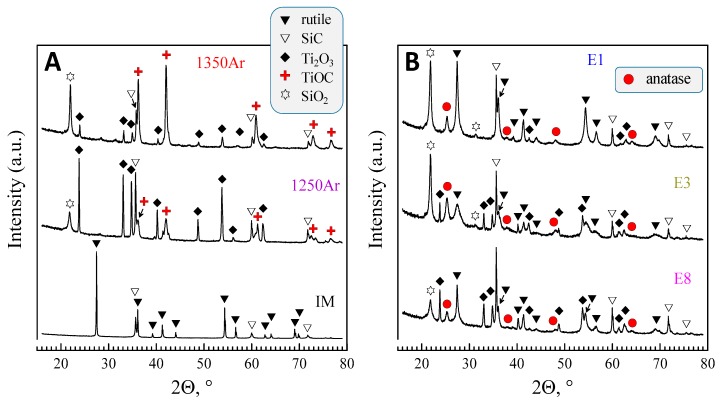
XRD patterns showing phase evolution from initial powder to pre-reduced (**A**) and post-oxidised samples (**B**).

The XRD results obtained for the samples processed in Ar atmosphere are shown in Figure 2. The results depict significant changes in the phase composition as compared to the initial mixture. The formation of at least three new phases, including reduced titanium oxide (Ti_2_O_3_, ICDD PDF #04-005-8760), titanium oxycarbide (TiOC, ICDD PDF #04-002-5443), and silicon oxide (SiO_2_, ICDD PDF #04-008-7640) was observed.

The results of the XRD quantification are given in Table 4.

In this table, the quantitative results in wt%. obtained by the Rietveld refinement of the XRD data were converted to mol% in order to better illustrate the reaction stoichiometry. It should be noticed that the nominal molar Ti/Si ratio of the initial mixture (1:1) is kept at an acceptable level in the results of the XRD quantification. As an example, this ratio corresponds to 0.97, 1.09, and 0.98 in the case of the samples 1250Ar, 1300Ar, and 1350Ar, accordingly. The latter is a good confirmation of the quality of the XRD quantification and its relevance for the analysis of phase transformations observed in the present work.

In general, assuming the observed phase composition, the reaction between titania and silicon carbide can be represented as follows:

(1) $$3{\mathrm{TiO}}_{2}+\mathrm{SiC}\to {\mathrm{Ti}}_{2}O_{3}+\mathrm{TiOC}+{\mathrm{SiO}}_{2}$$

However, this only describes the partial conversion of silicon carbide, and should yield about 40 mol% of residual SiC, 20 mol% Ti_2_O_3_, 20 mol% TiCO, and 20 mol% SiO_2_; this is only close to the results obtained at 1250 °C. Thus, one should consider the uptake of residual oxygen from the nominally inert Ar atmosphere (about 10 ppm), or due to imperfect sealing, possibly combined with slight carbon excess (or Si:C < 1), as often found in β-SiC, and also suggested by mol% SiO_2_ < mol% TiOC. Thus, one may assume a generic reaction:

(2) $${\mathrm{TiO}}_{2}+\left(1-2y\right){\mathrm{Si}}_{1-\delta }C+\left\{0.5-1.5y-\delta \left(1-2y\right)\right\}O_{2}\;\to {\mathrm{yTi}}_{2}O_{3}+\left(1-2y\right)\mathrm{TiOC}+\left(1-2y\right)\left(1-\delta \right){\mathrm{SiO}}_{2}$$

Equation (2) may describe departure from Equation (1), when y ≈ 1/3, and convergence to:

(3) $${\mathrm{TiO}}_{2}+{\mathrm{Si}}_{1-\delta }C+\left(0.5-\delta \right)O_{2}\to \mathrm{TiOC}+\left(1-\delta \right){\mathrm{SiO}}_{2}$$

mainly at the highest T_Ar_ temperature, as indicated by the prevailing formation of TiOC and drastic decrease of the fraction of Ti_2_O_3_ in the sequence TiCO > SiO_2_ >> Ti_2_O_3_.

**Figure 3:**
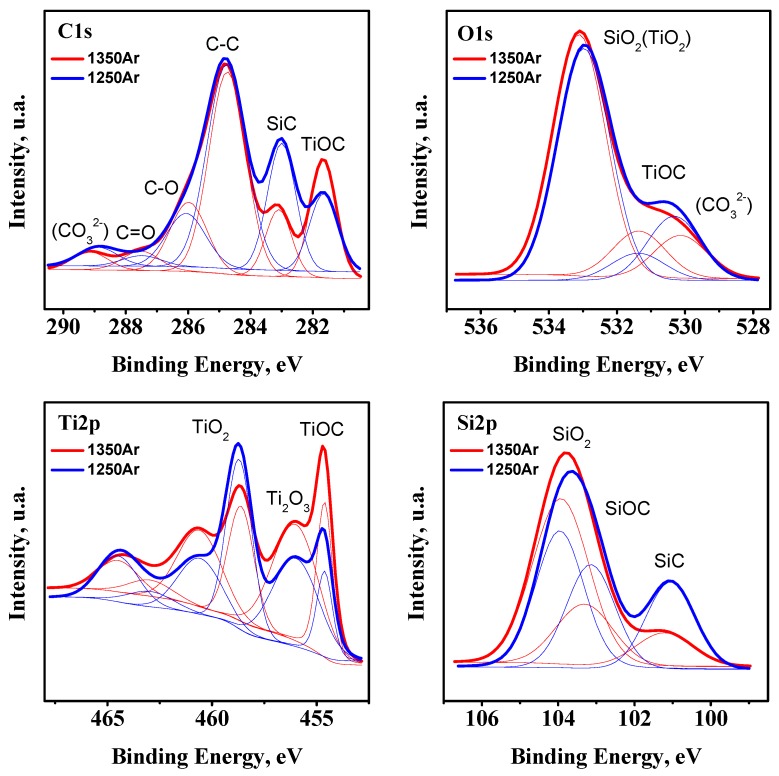
XPS of the representative pre-reduced samples in C 1*s*, O 1*s*, Ti 2*p*, and Si 2*p* core regions. The experimental data and simulated spectra are presented by thick and thin lines, accordingly.

Additional XPS studies of the pre-reduced samples were performed in order to confirm the XRD observations (Figure 3).

Since the XPS studies were performed for powdered samples produced under ambient air conditions, the C 1*s* core level spectrum shows the presence of adventitious carbon species, which correspond to C-C, C-O, and C=O bonds, with binding energies (BE) ~284.7, ~286.0, and 287.5 eV, and carbonates (CO_3_^2−^) (BE ~289.0 eV) and with the corresponding O 1*s* contribution at ~530.2 eV due to carbonates. The shape of the Ti 2*p* core level spectrum suggests the presence of a significant fraction of TiO_2_, along with the expected Ti_2_O_3_ (BE for Ti 2*p*_3/2_ ~458.6 and 456.0 eV, respectively) [28]. In addition to titanium oxide species, the results confirm the presence of TiOC, with a BE of ~454.7 eV for Ti 2*p*_3/2_ and a contribution at ~281.7 eV in the C 1*s* core level spectrum [29]. Since XPS is a surface characterisation technique, the presence of a significant fraction of TiO_2_ may arise from the partial surface oxidation of the powders before analysis. However, even more considerable uncertainties are expected from strong chemical inhomogeneity of the pre-reduced samples, including the presence of core-shell structures discussed below, with the surface shell composed mostly of silica. Further SiO_2_ and SiC species are also ascribed to BE for contributions in the Si 2*p* spectrum at ~103.9 and ~101.2 eV, respectively. As an example, an attempt to quantify the SiO_2_/SiC molar ratio from the XPS data gives the overestimated values of 3.7 and 9.1 for 1250Ar and 1350Ar samples, respectively, as compared to the data in Table 4 based on the results of the bulk XRD analysis. Still, in the latter case this ratio might be, in turn, partially underestimated due to possible amorphous structures of the silica shells. However, the results of XRD analysis appear to show a rather relevant picture of the phase composition, confirmed by the Ti/Si ratio close to the nominal 1:1 (Table 4). The slight asymmetry of the main Si 2*p* peak suggests the presence of the SiOC phase (BE of ~103.2 eV for Si 2*p*), not detected by the XRD, but a likely amorphous or intermediate product SiC_x_O_y_ between initial SiC and SiO_2_ phases [30], similarly to the case of TiOC.

The formation of titanium oxycarbide by carbothermal reduction of titania in inert or CO-containing atmospheres at the temperatures close to those employed in this work was previously observed in many studies (for example, [31,32]). In agreement with the cited papers, higher temperatures of the treatments in Ar increase the fraction of TiOC formed, the corresponding fraction of SiC decreases on the temperature increase. The deviations of TiOC to SiO_2_ molar ratio from 1:1, predicted in accordance with Equation (1), can be attributed to the partial oxidation of silicon carbide by oxygen residues presented in Ar and the possible presence of the amorphous phase. In general, the results suggest that the extent of the reaction between rutile and silicon carbide can be well controlled by sintering conditions. Although the time of Ar treatments was not varied in this work, kinetic control of this reaction is expected to allow flexible tuning of the phase composition and residual amount of SiC phase.

The pre-reduction step in Ar atmosphere allowed one to attain two prerequisites for further anatase formation and stabilization, aiming at tunable photocatalyst compositions. Firstly, both Ti-containing phases formed at pre-reduction (TiCO and Ti_2_O_3_) are capable of forming TiO_2_ on oxidation, either in the form of rutile or anatase, or as a co-existence of these polymorphs by post-oxidation at intermediate temperatures. Secondly, the presence of silica is expected to stabilize the fraction of anatase, in accordance with the results [22,23]. Thus, relatively mild post-oxidation conditions were selected, including the temperatures in the range 400–500 °C and different times. The selected XRD patterns of the oxidised samples are shown in Figure 2B.

**Figure 4:**
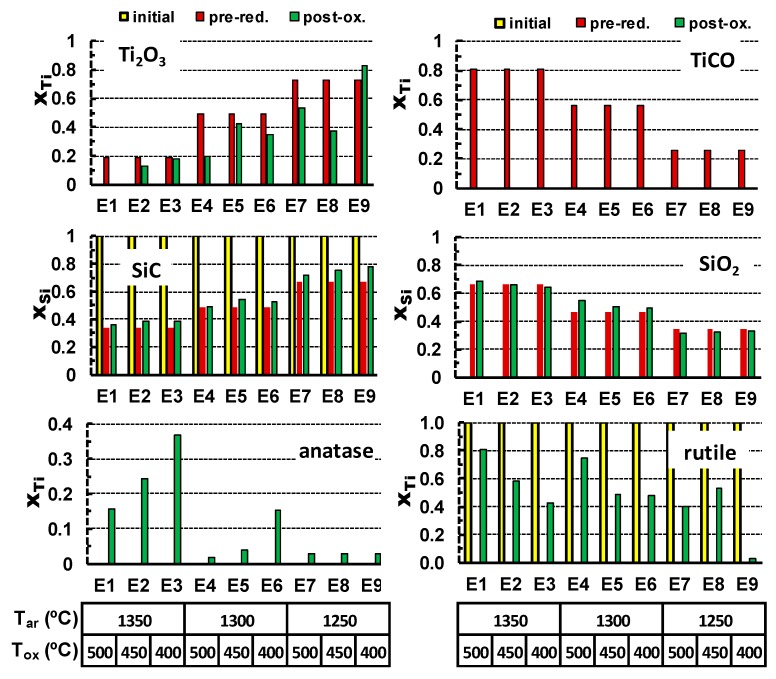
Evolution of relative contents of different phases (in terms of molar fractions of Ti or Si) from the initial precursors, through the re-reduction stage and after post-oxidation. Relevant temperatures of pre-reduction in Ar and subsequent oxidation in air are also shown.

The oxidation results in the vanishing of titanium oxycarbide phase and formation of both anatase and rutile polymorphs of TiO_2_. At the same time, Ti_2_O_3_ is still present in the samples after oxidation and disappears only in extreme oxidising conditions as 500 °C for 25 h. Corresponding results of the XRD quantification are also shown in Table 4, and guidelines for relevant reactions are shown in Figure 4. In this case, molar quantities of anatase, rutile (Y-axis), and other phases are converted to corresponding fractions of Ti or Si, to follow gradual changes from the initial precursors, through pre-reduction and the after post-oxidation. These results show the impact of TiCO on final yield of anatase after post-oxidation, combined with the opposite effect of the increase in oxidation temperature, which favours conversion to rutile. Note also that the residual content of SiC after the pre-reduction stage remains nearly unchanged upon oxidation at temperatures in the range 400–500 °C. The contents of SiO_2_ also remain nearly unchanged during the oxidation stage.

Other useful guidelines for the effects of processing conditions on the phase composition of the oxidised samples can be obtained from assessing the results of Taguchi experimental planning (Table 5).

The impact of each parameter was analysed using the linear regression model:

(4) $$\mathrm{mol}\%\left({\mathrm{Ti}}_{2}O_{3}/\mathrm{anatase}/\mathrm{rutile}/{\mathrm{SiO}}_{2}\right)=\alpha \cdot T_{\mathrm{Ar}}+\beta \cdot T_{\mathrm{ox}}+\gamma \cdot t_{\mathrm{ox}}+\theta$$

The correlation matrix and the values of regression coefficients obtained for the post-oxidised samples suggest the following trends. The processing conditions in Ar, which result in pre-reduction of titania, are of special importance for the anatase formation. Namely, a higher pre-reduction temperature enhances the yield of TiCO, which facilitates the formation of anatase by subsequent oxidation at much lower temperatures. Again, this correlates well with the trends in the formation of titanium oxycarbide, which, for the selected post-oxidising conditions, appears to be the main precursor for the anatase formation (Table 4 and Figure 4). The negative value of the correlation factor between the Ar-processing temperature and contents of Ti_2_O_3_ seems counterintuitive. One expects rather an increase in Ti_2_O_3_ concentration on increasing this temperature. However, this can be explained by the fact that the higher Ar processing temperature rather facilitates the formation of TiOC as compared to Ti_2_O_3_ (Table 4).

Upon oxidation, TiOC is fully converted to anatase/rutile even at the lowest oxidation temperature/time, while the Ti_2_O_3_ residuals still remain and thus suggest this type of apparent correlation. A higher oxidising temperature (T_ox_) appears favourable for the rutile formation as compared to anatase. The main parameter affecting the amount of SiO_2_ in the post-oxidized samples is the Ar-processing temperature, as indicated by the corresponding correlation factor above 0.98, while the factors for T_ox_ and t_ox_ are close to zero, indicating a very weak correlation with these parameters. The oxidation kinetics may be illustrated by the t_ox_ parameter, showing a moderate relevance of the oxidation time for the formation of rutile and, probably, for vanishing Ti_2_O_3_ oxide.

### 3.2. Microstructural Evolution

**Figure 5:**
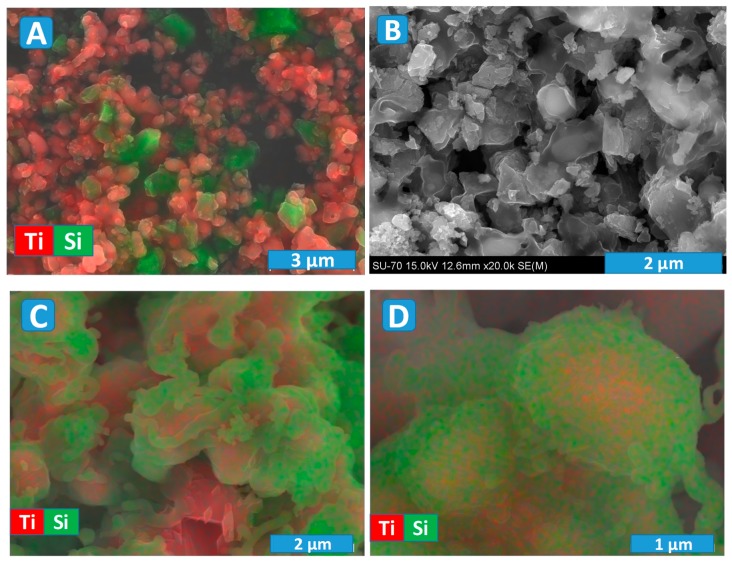
Combined SEM/EDS images for IM (**A**), 1350Ar pre-reduced ceramics (**B**) and 1300Ar (**C**,**D**) powders, showing the formation of core-shell structures.

A typical microstructure of the IM powder and corresponding distribution of the titania and silicon carbide particles are presented in Figure 5A. The particles size is similar to that for initial precursor powders, where SiC is mostly of micrometric size, while TiO_2_ rutile particles are of submicrometric or even nanometric (agglomerated) size. Indeed, the silicon carbide is well-known as a very hard material, and it was impossible to reduce its particle size by the milling procedure employed in the present work. Still, the used milling allows sufficient homogenisation, as it follows from the chemical analysis map (Figure 5A).

An example of the microstructure of the ceramic samples processed under Ar atmosphere is shown in Figure 5B. These results again evidence a significant porosity, which is expected to minimise oxygen diffusion limitations during following the post-oxidation step. The observed microstructures suggest the presence of a noticeable interaction between initial composite components, in agreement with the XRD results (Figure 2) discussed above. In particular, one can observe the formation of core-shell-like particles with a lighter core surrounded with a darker shell (Figure 5B). Thus, the prospective photocatalytic properties might be affected by microstructural features of the prepared materials, in addition to the phase composition.

More details regarding the composition of those core-shell particles can be revealed using EDS analysis, combined with SEM (Figure 5C,D). The images illustrate quite typical microstructural features in the sample, processed in Ar at an intermediate temperature of 1300 °C. The shell is enriched in silicon, and the corresponding morphology is very similar to a glassy/amorphous state. Since the silicon carbide particles have initially an absolutely different shape (Figure 5A), the shell is likely composed of SiO_2_, in agreement with the XRD results. Unfortunately, the used equipment and procedure do not allow tracking of the carbon contrast in the samples, since they required preliminary carbon deposition before the SEM/EDS characterisation. Thus, the core is composed of Ti_2_O_3_ and TiOC; the latter is rather likely formed closer to the surface in the space confined by the contact between initial TiO_2_ and SiC particles. Some Ti-rich particles have the surface clear from SiO_2_ (Figure 5C), while other particles look completely blocked (Figure 5C,D). Such blocking may impede oxidation, which is necessary for the formation of anatase-containing samples from Ar-processed materials. On the other hand, it could facilitate very delicate, kinetically controlled oxidation, which might be rather suitable for anatase formation and nanostructuring.

**Figure 6:**
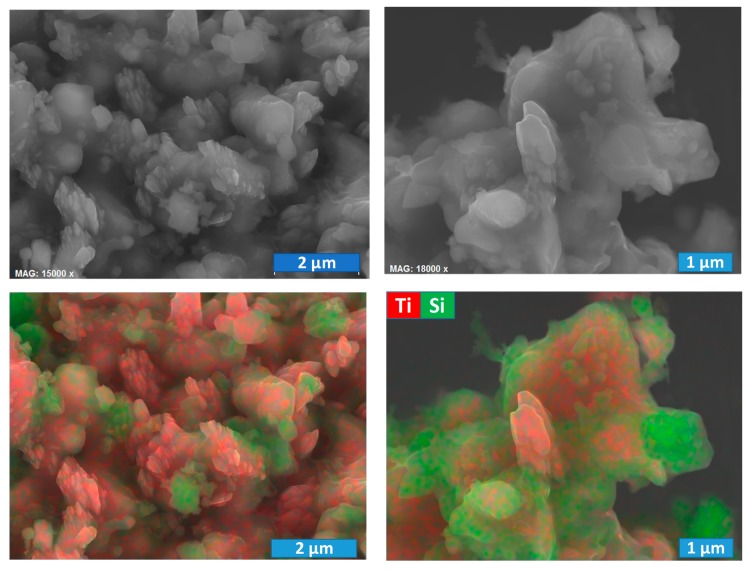
Combined SEM/EDS images for post-oxidized E4 powdered sample, showing the destruction of core-shell structures.

The microstructural studies performed for post-oxidised samples show the formation of more open structures (Figure 6), while part of the Ti-rich particles still has a SiO_2_ surface blocking layer.

Oxidation and the corresponding volume increase seem at least partially to destroy the SiO_2_ shell, leading to the formation of nanostructured titania polymorphs. The latter is especially well illustrated by the results obtained for the E4 oxidised sample, where columnar submicro- and nanosized titania-based structures appear to grow through the silica shell (Figure 6). Secondly, the observed microstructures of the oxidised samples look quite similar and do not allow revealing any tendencies with temperature/oxidation time based only on the microstructural studies. Thus, these results basically highlight that possible promoting effects on the photocatalytic performance, imposed by the formation of anatase and rutile under oxidising conditions, may be at least partially hindered by the surface blocking layers composed of SiO_2_. On the other hand, silica is known to have a promoting effect on the photocatalytic performance of titania, which involves the formation of local Ti-O-Si bonds [33]. In particular, a common strategy to benefit from this synergy includes the preparation of SiO_2_@TiO_2_ core-shell nanoparticles with enhanced activity [34]. Although the core-shell structures produced in the present work have, to a certain extent, an inversed architecture comprised of a titanium-rich core and silica shell, one still expects some synergistic effects towards photocatalysis based on TiO_2_-SiO_2_ interaction and formation of local Ti-O-Si linkages, invisible for the XRD technique. The corresponding photocatalytic tests were performed and described in the next section.

**Figure 7:**
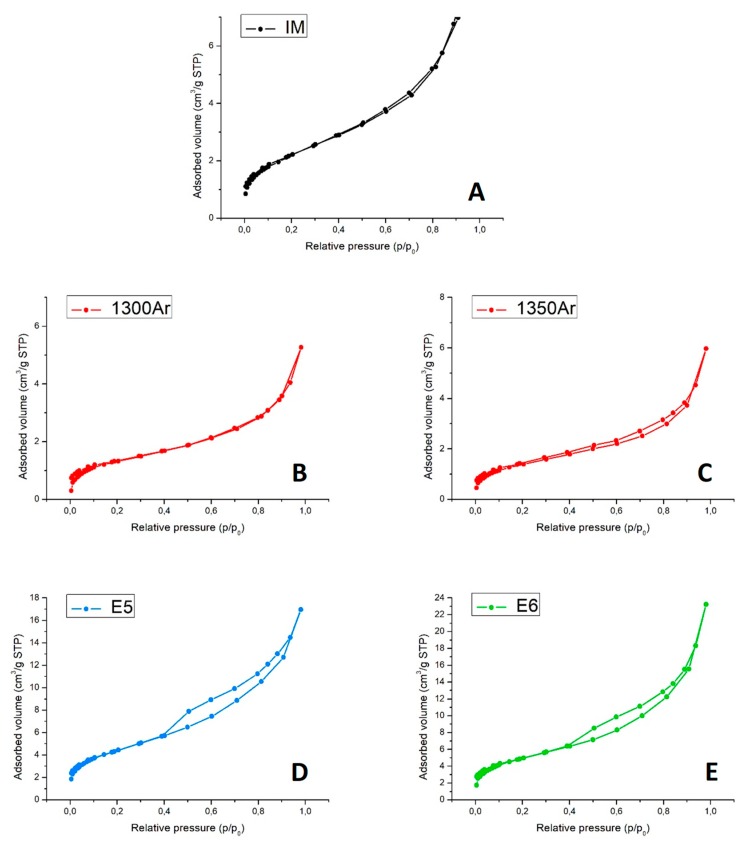
N_2_ adsorption (full symbols) and desorption (empty symbols) isotherms at −196 °C for IM (**A**), pre-reduced 1300Ar (**B**), 1350Ar (**C**) and post-oxidised E5 (**D**), and E6 (**E**) powdered samples.

Figure 7 presents the N_2_ adsorption–desorption isotherms at −196 °C.

The N_2_ adsorption–desorption isotherms of the initial mixture and reduced samples (Figure 7A–C) are of type II, which is characteristic of nonporous materials [35]. This is somewhat expected, taking into account the nature of the composite precursors’ powders with a (sub) micrometric particle size. Moreover, the reaction between titania and silicon carbide at elevated temperatures and formation of the core-shell structures are not likely to result in the appearance of the micro- and mesoporosity. On the contrary, the isotherms of oxidized samples (Figure 7D,E) present a hysteresis loop and are of type IV, which is characteristic of mesoporous sorbents. The results suggest that oxidising conditions induce the formation of some mesoporosity (pore width in the range 2–50 nm), very likely being promoted by the growth of nanostructured anatase.

Finally, Table 6 presents the values of the specific surface area and total pore volume.

Overall, the materials present a low total porosity. The results are in excellent agreement with the SEM/EDS studies, discussed above. The formation of core-shell structures results even in a decrease of the specific surface area. At the same time, the nanostructuring promoted by the delicate oxidation of the samples significantly enlarges the available surface, with the expected impact on the photocatalytic activity.

### 3.3. Photocatalytic Activity

**Figure 8:**
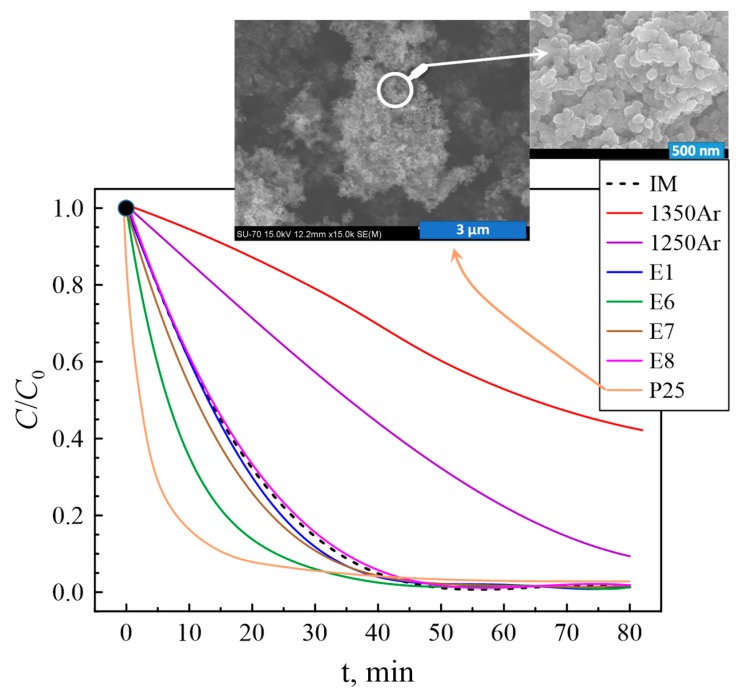
Photocatalytic degradation of methylene blue in the presence of nonmodified and redox-tuned sample powders. The insets show SEM micrographs of the P25 photocatalyst, used as a reference.

For the sake of comparison, the photocatalytic studies were performed for the initial IM powder, standard P25 TiO_2_-based photocatalyst as a reference and the samples converted to powder after processing under Ar and air atmospheres. During the photocatalytic process, the photo-generated electrons in the materials under study are transferred to the nearby methylene blue molecules and participate in the redox reactions resulting in the decomposition of methylene blue into H_2_O and CO_2_ [36,37]. Figure 8 presents a C/C_0_ plot against irradiation time for several selected compositions, where *C_0_* is the initial concentration after dark conditions stage and *C* is the concentration of methylene blue at different test times.

The initial IM powder shows a noticeable photocatalytic activity provided by the presence of rutile. As expected, the fastest photodegradation of the methylene blue was expectedly observed in the case of the standard P25 TiO_2_ powder, containing anatase and rutile phases in a ratio of about 4:1. As an example, the insets in Figure 8 show the typical microstructure of P25 particles, with a high degree of nanostructuring and obviously higher surface area as compared to the Ar-processed and post-oxidised samples produced in this work (Figure 5 and Figure 6). The Ar-processed nonoxidised samples, containing Ti_2_O_3_ and TiOC, demonstrate the lowest rate of photocatalytic degradation. Based on the results on specific surface areas calculated from the BET equation (Table 6), this behaviour can be attributed to the blocking of the active area, as also revealed by combined SEM/EDS studies (Figure 5C,D). It should be noticed that, although the presence of significant amount of Ti^3+^ species may facilitate the recombination of the charge carriers and, thus, effectively suppress the photodegradation, the photoactivation process for TiOC and Ti_2_O_3_ phases may be actually shifted to the visible light [28,38,39], while the studies in the present work were performed using an ultraviolet lamp. This means that Ar-processed samples may still possess some photocatalytic activity under visible light, although microstructural constraints imposed by the presence of silica shells are likely to counterbalance this improvement.

On the contrary, the major part of the post-oxidised samples promotes faster photodegradation of the methylene blue as compared to the initial IM mixture, in agreement with the tendencies observed for the phase composition and specific surface area. The best performance was observed for the E6 sample, prepared by processing in Ar at an intermediate temperature (1300 °C) and post-oxidised at the lowest temperature (400 °C) for the longest oxidation time of 25 h. The latter may indicate that the range of processing conditions under oxidising atmosphere should be further optimised taking into account possible kinetic limitations if aiming at further improvement of the photocatalytic properties.

Finally, the impacts of the processing conditions on photocatalytic activity of the post-oxidised samples were analysed based on the values of the apparent rate constant of the first-order kinetics (*K_ap_*) and the half-life time of photocatalytic degradation (*t_1/2_*), expressed by Equations (5) and (6), respectively:

(5) $$\ln\left(C/C_{0}\right)=K_{ap}\times t$$

(6) $$t_{1/2}=\frac{\ln\left(2\right)}{K_{ap}}$$

The calculated values of the rate constant and half-life time are given in Table 7, as well as the linear regression correlation coefficient (R^2^).

The highest *K_ap_* value of 0.0893 min^−1^ and lowest *t*_1/2_ of 7.8 min were observed for the E6 sample. On the other hand, in the same testing conditions, the performance of industrial P25 photocatalyst is roughly ~1.6 better than that for the E6 sample. It should be noticed that the actual fraction of the photocatalytically-active species is significantly higher in P25, due to the presence of silica-based species in the post-oxidised samples. However, a rather important issue is that the surface area of the best-performing E6 sample is ~2.7 times lower than for P25. Thus, in terms of the area-specific photocatalytic activity, several post-oxidized materials show comparable values or even outperform the P25 photocatalyst. These results show an obvious potential for designing the performing titania-based photocatalysts using the proposed approach. 

Table 8 presents the values of correlation factors for the effects of processing conditions on photocatalytic activity of the post-oxidised samples.

Surprisingly, but these results predict the major impact of the oxidation time on *K_ap_* and *t*_1/2_ values, while a similar analysis for the phase composition (Table 5) suggests that the amounts of Ti_2_O_3_, anatase, rutile, and SiO_2_ phases are determined by the Ar-processing and post-oxidation temperatures rather than by *t_ox_*. It should be noticed that the maximum difference in the measured photocatalytic activities between E1, E2, E3, E7, E8, and IM samples corresponds to ~30%. Such a moderate variable variation may increase the errors associated with the Taguchi plan analysis, while the relevance of the *t_ox_* may be simply conditioned by the fact that the highest photocatalytic performance was observed for the sample oxidized for 25 h. In addition to the phase composition, the photocatalytic activity is strongly influenced by the microstructural features and availability of the surface for the reaction, which, in turn, can be affected by the formation of core-shell particles. As an example, while in several cases the formation of anatase/rutile mixture by post-oxidation is expected to be advantageous for the photodegradation reaction rate as compared to the initial mixture, partial blocking of the active surface by silica may impair this positive effect. Additional studies are required to confirm the relevant mechanisms behind the observed effects of the processing conditions on the phase composition and photocatalytic performance. Still, in terms of the degradation efficiency compared to irradiation time, the best photocatalytic activity observed for the E6 redox-tuned sample is comparable or even exceeds the corresponding performance for several nanostructured photocatalysts found in the literature data (Table 9).

Although some differences may originate from the variation in testing methodology, the results clearly demonstrate the potential of the proposed concept for designing titania-based multifunctional photocatalysts by controlled redox reactions. However, further tests involving different dyes/contaminants, behaviour under visible light irradiation and microwave heating, and reusability assessment are essential on the way towards practical applications.

## 4. Conclusions

The prospects for designing multifunctional titania-based photocatalysts by controlled redox reactions in a TiO_2_-SiC mixture were assessed. The research has demonstrated a potential alternative route towards efficient TiO_2_-based photocatalysts using conventional raw materials. The selected processing approach included a partial pre-reduction of titania by silicon carbide in inert argon atmosphere, intending to produce precursors capable of forming anatase in the oxidising conditions. This was followed by delicate oxidation under air conditions to produce TiO_2_, both in the form of rutile and anatase. A Taguchi experimental planning was used to evaluate the effects of processing conditions, namely, the temperature of processing in Ar (*T_Ar_*–1250, 1300, and 1350 °C), the post-oxidation temperature (*T_ox_*–400, 450, 500 °C), and time (*t_ox_*–1, 5, and 25 h) on the phase composition and photocatalytic activity. The main conclusions of the work are the following:

Selected range of processing conditions in Ar and air atmospheres allowed controlled interaction between the initial composite components, leading to the formation of partially reduced titanium-containing compounds, which can be then oxidised to form the rutile/anatase mixture. The final composite also retained a fraction of the SiC phase, which is required for thermal co-activation functionality.

Higher temperatures of the treatment in Ar (*T_Ar_*) promoted the formation of titanium oxycarbide TiOC, which acted as the main precursor for the anatase formation in post-oxidised samples. The analysis of correlation effects in the selected Taguchi experimental plan has shown that a higher oxidising temperature (*T_ox_*) is more favourable for the rutile formation as compared to anatase. The effects of the treatment temperatures both in Ar and air were shown to be the most important for the phase composition, whilst the oxidation time was only moderately relevant.

Microstructural studies have revealed the formation of core-shell microstructures in the case of the samples processed in Ar, with the shell composed of silica and the core rich in a titanium-containing species. This microstructural evolution resulted in the limited surface area in these samples. Post-oxidation resulted in at least partial destruction of the SiO_2_ shell, leading to the formation of nanostructured titania polymorphs and a noticeable increase in the surface area.

The intermediate phase TiOC oxidised readily in air, even at 400 °C; this raises good prospects for further optimization of the final oxidation stage at even lower temperatures, possibly in pure O_2_, to seek higher anatase fractions and improved nanostructuring.

The phase composition and microstructural evolution in the post-oxidised samples imposed a noticeable effect on the photocatalytic performance. The activity of the best-prepared material, expressed in the apparent rate constant, equal to 0.089 min^−1^, was only ~1.6 times lower than the corresponding activity of the nanostructured industrial P25 photocatalyst possessing a 2–3 times larger surface area. The results of Taguchi analysis suggested oxidation time (*t_ox_*) as a most impactful parameter of the selected processing conditions on the photocatalytic activity.

## Figures and Tables

**Table 1:** Variable processing parameters and their levels.

	Variables		
Level	Pre-Reduction Temperature (*T_Ar_*), °C	Post-Oxidation Temperature (*T_ox_*), °C	Post-Oxidation Time (*t_ox_*), h
1	1350	500	25
2	1300	450	5
3	1250	400	1

**Table 2:** L_9_ orthogonal array of the processing conditions used in Taguchi planning.

Experiment/Sample Denomination	*T_Ar_*, °C	*T_ox_*, °C	*t_ox_*, h
E1	1350	500	25
E2	1350	450	5
E3	1350	400	1
E4	1300	500	5
E5	1300	450	1
E6	1300	400	25
E7	1250	500	1
E8	1250	450	25
E9	1250	400	5

**Table 3:** Denominations of other samples used for comparative studies.

Sample Denomination	*T_Ar_*, °C
IM	initial mixture
1250Ar	1250
1300Ar	1300
1350Ar	1350

**Table 4:** Results of quantification of the phase composition of the redox-tuned samples.

Phase	Pre-Reduced Samples, mol%			Post-Oxidised Samples, mol%								
	1250Ar	1300Ar	1350Ar	E1	E2	E3	E4	E5	E6	E7	E8	E9
Ti_2_O_3_	22.2	13.9	4.9	−	3.4	4.6	5.1	11.9	9.5	15.4	10.2	26.2
TiO_2 (rutile)_	−	−	−	40.1	30.1	22.4	39.4	27.1	26.2	23.3	29.2	1.7
TiO_2 (anatase)_	−	−	−	7.8	12.6	19.2	0.9	2.1	8.4	1.6	1.5	1.8
SiC	40.9	27.7	17.6	17.9	19.8	20.1	26.0	30.6	28.6	41.6	41.3	49.4
SiO_2_	21.1	26.5	34.9	34.1	34.1	33.8	28.6	28.3	27.3	18.1	17.8	20.9
TiCO	15.8	31.9	42.6	−	−	−	−	−	−	−	−	−

**Table 5:** Correlation matrix for the effects of Ar-processing temperature (T_Ar_), post-oxidation temperature (T_ox_), and time (t_ox_) on mol% of the Ti-containing species in the oxidized samples, correlation coefficient (K_r_), and corresponding coefficients (α, β, γ and Θ) of the linear regression model described by Equation (4).

mol%	Correlation Matrix			Kr	α (T_Ar_)	β (T_ox_)	γ (t_ox_)	Θ
	T_Ar_	T_ox_	t_ox_					
% Ti_2_O_3_	−0.808	−0.365	−0.278	0.993	−0.146	−0.066	−0.195	2301
% anatase	0.787	−0.432	−0.061	0.995	0.116	−0.064	−0.035	−115
% rutile	0.491	0.675	0.340	0.874	0.127	0.175	0.343	−222
% SiO_2_	0.983	−0.024	−0.053	0.985	0.151	−0.004	−0.032	−167
% SiC	−0.961	−0.162	−0.082	0.983	−0.248	−0.042	−0.082	373

**Table 6:** Specific surface area calculated using the BET equation (S_BET_) and total pore volume (V_T_), estimated from Gurvitch’s rule.

Sample	S_BET_ (m^2^/g)	V_T_ (cm^3^/g)
E6	18.3	0.0363
E5	16.1	0.0265
1300Ar	4.8	0.0082
1350Ar	5.0	0.0093
IM	7.7	0.0189
P25	48.6	-

**Table 7:** Comparison between the values of K_ap_ and the half-life decomposition time for the reference and post-oxidised samples.

Photocatalytic Parameters	IM	P25	E1	E2	E3	E4	E5	E6	E7	E8	E9
*K_ap_* × 10^2^, min^−1^	7.18	14.33	7.53	7.03	7.37	6.54	5.71	8.93	7.61	6.81	6.75
*t*_1/2_, min	9.7	4.8	9.2	9.9	9.4	10.6	12.1	7.8	9.1	10.2	10.3
R^2^	0.987	0.950	0.983	0.990	0.978	0.992	0.960	0.987	0.994	0.986	0.994

**Table 8:** Correlation matrix for the effects of processing conditions on the photocatalytic activity of post-oxidised samples.

Parameter	T_Ar_	T_ox_	t_ox_
K_ap_ (methylene blue)	0.124	−0.223	0.503
*t*_1/2_	−0.133	0.170	−0.480

**Table 9:** Comparison of the observed photocatalytic performance with the literature data obtained under ultra violet (UV) irradiation.

Photocatalyst	Degradation Efficiency (1−*C*/*C*_0_), %	Irradiation Time, min	Initial MB Concentration, mg/L	Initial Photocatalyst/MB Weight Ratio	Reference
E6	80	16	10	50	this work
E6	90	23	10	50	this work
P25	80	8	10	50	this work
P25	90	16	10	50	this work
TiO_2_ NPs/CdSe nanocrystals platform	68	75	10	59	[40]
TiO_2_ NPs (hydrothermal route)	80	76	10	10	[41]
TiO_2_ P25	80	14	10	100	[42]
TiO_2_ P25	90	21	10	100	[42]
sol-gel TiO_2_ (7 wt% Fe_2_O_3_) NPs	80	31	3.2	3.1	[16]
sol-gel TiO_2_ (7 wt% Fe_2_O_3_) NPs	90	42	3.2	3.1	[16]
TiO_2_/ZnS NPs	80	7	20	25	[43]
TiO_2_/ZnS NPs	90	9	20	25	[43]
TiO_2_/0.16 mol% Cu_2_O NPs	80	18	10	50	[17]
TiO_2_/0.16 mol% Cu_2_O NPs	90	25	10	50	[17]
TiO_2_ NPs/ coconut shell activated carbon nanocomposites	80	16	50	20	[44]
TiO_2_ NPs/ coconut shell activated carbon nanocomposites	90	25	50	20	[44]
